# Role of Andaman and Nicobar Islands in eddy formation along western boundary of the Bay of Bengal

**DOI:** 10.1038/s41598-019-46542-9

**Published:** 2019-07-12

**Authors:** A. Mukherjee, Abhisek Chatterjee, P. A. Francis

**Affiliations:** Indian National Centre for Ocean Information Services (INCOIS), Hyderabad, India

**Keywords:** Physical oceanography, Physical oceanography

## Abstract

Eddies along western boundary of the Bay of Bengal (WBoB) play an important role in regulating regional climate and marine productivity of the north Indian Ocean. In this paper, role of Andaman and Nicobar islands (ANIs) in the formation of eddies along the WBoB is studied using an ocean general circulation model. Our analysis shows that, in the absence of ANIs, there is a significant reduction in the total number of mesoscale eddies in this region. The impact is particularly evident for the cyclonic eddies as a reduction of ~50% can be noticed in the absence of the islands. In contrast, influence of ANIs on anticyclonic eddies is not homogeneous in the WBoB; while absence of ANIs significantly increases anticyclonic eddies in the central part of the WBoB, a decrease can be noticed in the southern part. We further show that the reduction in number of cyclonic eddies along the WBoB is primarily driven by reduced baroclinic and barotropic instabilities. This process is more conspicuous during winter (October–January) season compared to summer (June–September) and spring (February–May) seasons.

## Introduction

Eddies are known as turbulent rings which trap cold (cyclonic eddy) or warm (anticyclonic eddy) water at their centre and isolate it from the mean flow. These eddies play an important role in regional and global climate^1^ by transporting heat and salt in the world ocean. Eddies are also important for regulating marine productivity. Previous studies^2–4^ suggest that cyclonic eddies upwell subsurface water in its core, which enhances nutrient concentrations in the upper surface layer of the water column and help in increasing productivity. Moreover, information on location of eddies and their characteristics are important for other marine activities such as shipping, Oil and natural gas explorations, etc. Therefore, understanding the variability of oceanic eddies and their accurate numerical simulation are key to the economic sustainability of the coastal regions.

Circulation in the Bay of Bengal (BoB) are known for eddy–mean flow interactions driven by the strong East India Coastal Current (EICC) and spatial gradient in the density distribution^5–7^. Owing to the westward propagation of mesoscale eddies embedded within the large scale planetary Rossby waves, circulation in the western part of the BoB (WBoB) are strongly dominated by eddy–mean flow interactions compared to the eastern part^6^. In fact, intense eddy activities are observed in the entire BoB during all the three dominant seasons viz., winter (October–January), summer (June–September) and spring (February–May)^6–8^.

Ocean internal instability, local Ekman pumping and remote response from the equatorial Indian Ocean (EIO) are also believed to play important roles for the eddy variability in the BoB^9^. As these equatorial Kelvin waves reaches the coast of Sumatra, bifurcate into coastal Kelvin waves. Northern branch of this coastal Kelvin wave then propagate along the eastern boundary of the BoB and subsequently radiate Rossby waves into the interior bay^10^. It is shown that these Rossby waves generate instability and promote the formation of eddies owing to the interactions with the coastal currents of the WBoB^6,7^. Several recent studies^6,7,9,11^ suggest that these Rossby wave interactions predominantly generate baroclinic instabilities. On the other hand, barotropic instabilities are reported to be relatively weaker in this region.

The eastern part of the BoB is divided by a narrow chain of islands, known as the Andaman and Nicobar islands (ANIs) which separate a small basin, called Andaman Sea, from the rest of the bay. A recent study by Chatterjee *et al*.^10^ showed the importance of this island chain in modifying the circulation of the central BoB and the EICC. Later, Cheng *et al*.^12^ showed that the Rossby waves, radiated out from the coastal Kelvin wave, get significantly altered by the ANIs and increase eddy activity in the central BoB. But, their analysis was restricted only in the central and eastern BoB (east of 85°E). However, the processes that drive the eddy activity in the WBoB, a region of economic significance for India and other neighboring countries, remains unexplored.

In this manuscript, we explored the significance of ANIs for the formation of eddies over the BoB with a special emphasize on the WBoB region. Note here that the domain of our analysis for this study is restricted within 3–23°N and 78–98°E.

## Data and Models

We have used an eddy tracking algorithm for eddy identification based on Mason *et al*.^13^. Gridded altimeter Sea Level anomaly (SLA) from Archiving, Validation and Interpretation of Satellite Oceanographic (AVISO) data (https://www.aviso.altimetry.fr) has been used for observed eddy analysis. Before applying the eddy tracking algorithm, a 5-point Hamming window smoother is applied on the SLA field to remove high frequency noises. Then SLA contours are computed at 1-cm intervals within −100 cm to 100 cm range. In order to identify an eddy, closed contours are sequentially identified at each SLA intervals and analysed. Detailed discussion on this eddy tracking algorithm are available in Mason *et al*.^13^; however, a brief description is given in Section S1 for brevity. In this study, mesoscale eddies are identified with amplitude, radius and life cycle grater than 4 cm, 50 km and 28 days, respectively.

We have used an ocean general circulation model, known as Regional Ocean Modeling system (ROMS) version 3.6 developed by Rutgers University^14^. In ROMS, primitive equations are discretized based on C-grid, hydrostatic, Boussinesq and free-surface assumptions. Vertical grids are based on terrain following vertical sigma coordinate. Model domain is the Indian Ocean and extends between 30°–120°E and 30°S–30°N (Fig. S1). The horizontal resolution of the model is set to uniform 1/12°. There are 40 vertical levels, in which top 23 levels are within ~200 m of the water column where depth is ~1000 m. Detailed model set-up, numerical schemes and parametrization are discussed in Section S2 and in Jithin *et al*.^15^.

Model is forced by 6–hourly reanalysis atmospheric fields from Global Forecast System (GFS), obtained from National Centre for Medium Range Weather Forecasting (NCMRWF)^16^. More details about these forcing are available at http://www.ncmrwf.gov.in/gfs_report_final.pdf. The model simulation forced with complete physics and realistic atmospheric forcing is the closest solution to the observations and referred here as CR.

Further, in order to understand the contribution of ANIs, we have performed an ideal experiment by removing ANIs from the model bathymetry and henceforth will be referred as CR_NoANIs_. In this experiment, model topography and land mask associated with ANIs have been converted to ocean with depth values are filled using linear interpolation from the neighboring depth contours, keeping in mind that depth should be more than ~1000 m in the vicinity of the masked islands (Fig. S1). Moreover, in order to understand the role of ocean internal instability on the eddy genesis in the BoB, two additional experiments are carried out where the intraseasoanl variability from the wind forcing is removed by applying a 150-day low-pass based on fourth order Butterworth filter. While the experiment using complete bathymetry will be referred as CR′, the experiment with removed ANIs will be referred as ${C{\rm{R}}^{\prime} }_{{\rm{NoANIs}}}$.

## Results

**Figure 1:**
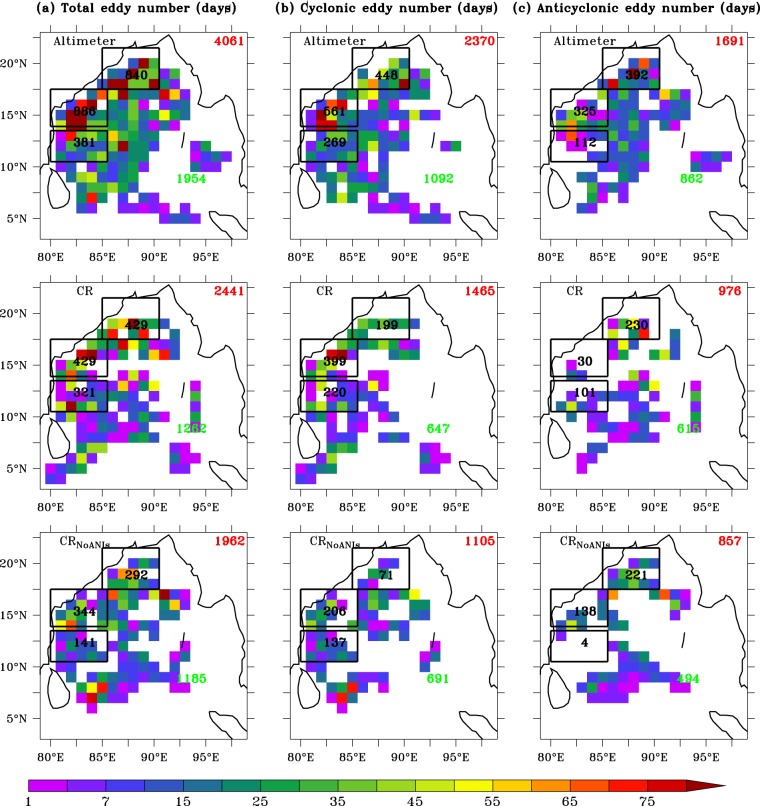
Comparison of number of eddies (1° × 1° in x–y) between altimeter and models (CR and CR_NoANIs_) for total (combination of cyclonic and anticyclonic) (**a**), cyclonic (**b**) and anticyclonic (**c**) number of eddies. The rectangular box between 80°–85°E/10°–13°N, 80°–85°E/14°–16°N and 85°–90°E/17°–21°N denotes SWBoB, CWBoB and NWBoB, respectively. Number mentioned in three rectangular boxes represent sum of number of eddies in the respective region during January 2011–December 2015. The number in red (green) denotes sum of number of eddies in the entire BoB (ResBoB). Comparison of eddy track and SLA between altimeter and models are shown in Figs S2 and S4 respectively. Comparison of number of eddies between CR′ and ${{\rm{C}}{\rm{R}}^{\prime} }_{{\rm{NoANIs}}}$ is shown in Fig. S6. Black contour represents land-sea masking based on Etopo20. Model domains is shown in Fig. S1.

Spatial maps of number of eddies derived from the altimeter SLA data for the period 2011–2015 shows dominance of cyclonic eddies (~58%) compare to anticyclonic eddies (~42%) in the BoB (78–98°E/3–23°N) (Fig. 1). It is evident that even within the WBoB, intensity of the eddies varies from south to north. In order to isolate and understand the mesoscale variabilities and to identify underlying processes, we further divided WBoB in three parts: northern (85–90°E/18–21°N), central (80–85°E/14–17°N) and southern (80–85°E/10–13°N) and henceforth will be referred as NWBoB, CWBoB and SWBoB, respectively. The rest of the BoB, i.e. the regions other than our region of interests (WBoB), will be referred as ResBoB, which includes central and eastern part of the BoB.

Model CR could simulate the observed eddy variability reasonably well, however underestimates the number of eddies compared to altimeter. CR simulates a total of 2441 (1465 cyclonic and 976 anticyclonic) mesoscale eddies in the BoB compared to 4061 eddies (2370 cyclonic and 1691 anticyclonic) observed in the altimeter data (Fig. 1a–c). A similar comparison can be seen for the WBoB, where model simulates 1179 eddies (818 cyclonic and 361 anticyclonic) compared to 2107 eddies (1278 cyclonic and 829 anticyclonic) in altimeter and for the ResBoB where model simulates 1262 eddies (647 cyclonic and 615 anticyclonic) compared to 1954 eddies (1092 cyclonic and 862 anticyclonic) observed in altimeter. CR also performed well in simulating observed track and life cycle of the eddies (Figs S2 and S3). Time-series analysis of SLA between CR and altimeter also shows a very high correlation (more than 0.5 in the WBoB) and near zero RMSE for the entire BoB even in the intraseasonal and seasonal time scale (Fig. S4).

In order to understand the role of ANIs on the eddy genesis in the WBoB, we have compared results from CR_NoANIs_ with CR. We find a significant reduction in number of eddies (~20%) in CR_NoANIs_ compared to CR for the entire BoB (Fig. 1a). Particularly, this reduction in number of eddies is much prominent for the WBoB compared to ResBoB: while in the WBoB the number of eddies decreased from 1179 to 777 (a reduction of ~35%), in the ResBoB, a marginal decrease from 1262 to 1185 i.e. a decrease of less than ~1% is observed. Further, we find that this observed decrease is primarily contributed by the decrease in cyclonic eddies compared to anticyclonic eddies (Fig. 1b,c). Notably, in the absence of ANIs, number of cyclonic eddies in the SWBoB, CWBoB and NWBoB decreases from 199, 399 and 220 to 71, 206 and 137, respectively i.e. a decrease of ~50% (from 818 to 414) in terms of total cyclonic eddies in the entire WBoB (Fig. 1b).

On the other hand, response of ANIs on the anticyclonic eddies is heterogeneous (Fig. 1c). Interestingly, in the absence of ANIs, anticyclonic eddies in the SWBoB almost got wiped out (101 to 4), but a significant increase is seen in the CWBoB (30 to 138). In contrast, the NWBoB does not show any major change in the number of anticyclonic eddies (231 to 221). Owing to this heterogeneity, overall in the WBoB, number of anticyclonic eddies remain relatively unchanged (361 to 363) between CR and CR_NoANIs_ experiments.

### Seasonal cycle

**Figure 2:**
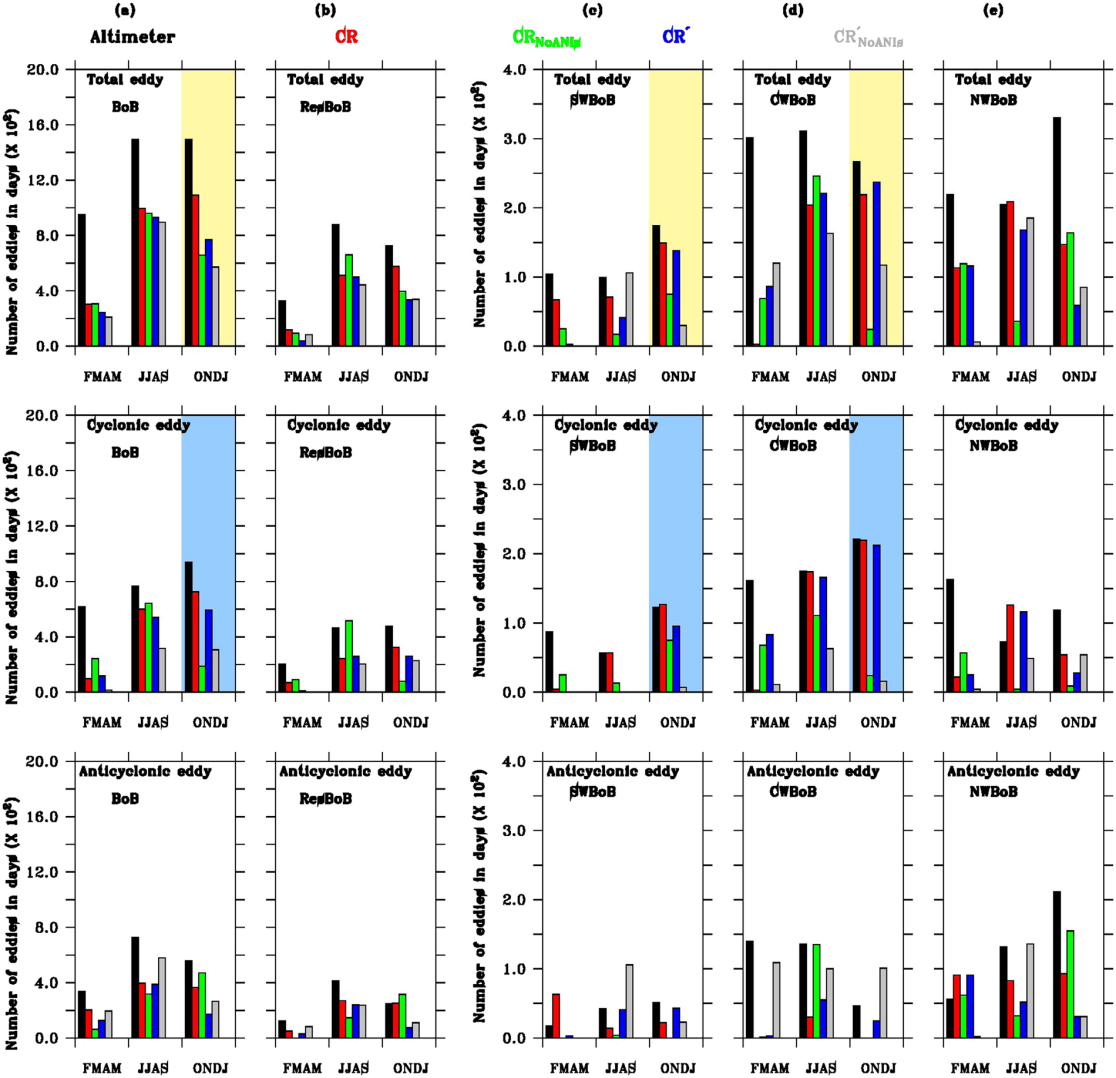
Histogram shows seasonal variations of number of eddies (spring, summer and winter) for total (combination of cyclonic and anticyclonic) (top panel), cyclonic (middle panel) and anticyclonic (bottom panel) number of eddies during January 2011–December 2015. The domain of histogram in every panel have been mentioned using BoB (**a**), ResBoB (**b**), SWBoB (**c**), CWBoB (**d**) and NWBoB (**e**). Black, red, green, blue and grey colour used in figure represents altimeter, CR, CR_NoANIs_, CR′ and ${{\rm{C}}{\rm{R}}^{\prime} }_{{\rm{NoANIs}}}$ simulations. Yellow (light blue) shaded region in top (middle) panel represents histogram diagram during winter for total (cyclonic) eddy. Date used for plotting this Figure is shown in Table 1.

In order to understand seasonal evolution of number of eddies in the BoB, we analysed our results for spring (February–May), summer (June–September) and winter (October–January) seasons. The definition of seasons are based on seasonal variability of the EICC discussed in Mukherjee *et al*.^17,18^. Note also that the seasonal means of the number of eddies are computed for the year 2011–2015 (Fig. 2a–e and Table 1). Both, altimeter and model CR, data set shows large seasonal variability in the BoB (Fig. 2). In altimeter, while the number of eddies are relatively high in summer (~38%) and winter (~38%), spring shows a relatively weaker activity (~24%). Model CR also shows similar high number of eddies as altimeter during summer and winter seasons and exhibits ~42%, ~46%, respectively. Note here that, while model does reasonably well to capture the seasonal number of eddies for summer (996) and winter (1091) seasons, the contribution of spring season is much smaller (301) in the model as opposed to altimeter. Possible reasons related to inability of our model in simulating number of eddies during spring season are discussed in the summary section.

**Table 1:** Number of eddies used in Fig. 2 for total eddy (TT; sum of cyclonic and anticyclonic), cyclonic (CC) and anticyclonic (ACC).

Eddy type	Spring TT	Summer TT	Winter TT	Spring CC	Summer CC	Winter CC	Spring ACC	Summer ACC	Winter ACC
Altimeter BoB	952	1493	1495	616	768	938	336	725	557
Altimeter ResBoB	328	878	724	205	463	475	123	415	249
Altimeter SWBoB	104	99	174	87	57	123	17	42	51
Altimeter CWBoB	301	311	267	161	175	221	140	136	46
Altimeter NWBoB	219	205	330	163	73	119	56	132	211
CR BoB	301	996	1091	98	600	725	203	396	366
CR ResBoB	118	512	576	69	243	325	49	269	251
CR SWBoB	67	71	149	4	57	127	63	14	22
CR CWBoB	3	204	219	3	174	219	0	30	0
CR NWBoB	113	209	147	22	126	54	91	83	93
CR_NoANIs_ BoB	305	960	659	242	643	188	63	317	471
CR_NoANIs_ ResBoB	92	661	396	92	515	80	0	146	316
CR_NoANIs_ SWBoB	25	17	75	25	13	75	0	4	0
CR_NoANIs_ CWBoB	69	246	24	68	111	24	1	135	0
CR_NoANIs_ NWBoB	119	36	164	57	4	9	62	32	155
CR′ BoB	243	929	768	117	540	595	126	389	173
CR′ ResBoB	38	499	334	9	258	260	29	241	74
CR′ SWBoB	3	41	138	0	0	95	3	41	43
CR′ CWBoB	86	221	237	83	166	212	3	55	25
CR′ NWBoB	116	168	59	25	116	28	91	52	31
${{\rm{C}}{\rm{R}}^{\prime} }_{{\rm{NoANIs}}}$ BoB	305	960	659	15	317	306	194	579	265
${{\rm{C}}{\rm{R}}^{\prime} }_{{\rm{NoANIs}}}$ ResBoB	83	442	339	0	205	229	83	237	110
${{\rm{C}}{\rm{R}}^{\prime} }_{{\rm{NoANIs}}}$ SWBoB	0	106	30	0	0	7	0	106	23
${{\rm{C}}{\rm{R}}^{\prime} }_{{\rm{NoANIs}}}$ CWBoB	120	163	117	11	63	16	109	100	101
${{\rm{C}}{\rm{R}}^{\prime} }_{{\rm{NoANIs}}}$ NWBoB	6	185	85	0	49	54	2	136	31

Detailed of models are described in Data and Model sections.

In the absence of ANIs (CR_NoANIs_), during winter season, a significant reduction in number of eddies is observed for SWBoB (149 to 75) and CWBoB (219 to 24), whereas a meager increase (147 to 164) is noticed in the NWBoB (Fig. 2c–e and Table 1). In contrary, during summer season, decrease in number of eddies is noticed in NWBoB (209 to 36) and SWBoB (71 to 17), while CWBoB show a marginal increase (204 to 246). This marginal increase of number of eddies may be associated with strong mean current at CWBoB during summer seasons, which prevents more eddy formation compared to SWBoB and NWBoB (Fig. S5 and also discussed in Mukherjee *et al*.^18^). Overall, in the absence of ANIs, both the seasons, summer and winter, show significant decrease in number of eddies across the WBoB. While in summer seasons, number of eddies decreases from 484 to 289 (i.e. ~40%), in the winter seasons, it decreases from 515 to 263 (i.e. ~50%). Further analysis suggests that this reduction in number of eddies during both summer and winter seasons are mainly contributed by reduction in cyclonic eddies and in fact, anticyclonic eddies slightly increases in the absence of ANIs. In the WBoB, cyclonic eddies decreases from 357 (400) to 128 (108) during summer (winter) seasons in the absence of ANIs. Whereas, anticyclonic eddies increases from 127 (115) to 171 (155) during summer (winter) seasons in the absence of ANIs.

### Role of ocean internal instabilities

We have found that more than 96% of mesoscale eddies detected from altimeter, CR and CR_NoANIs_ have life cycles between ~28 days to ~150 days (Fig. S3). This suggests the significance of above intraseasonal time scales in mesoscale eddy formation in the BoB. In order to remove the contribution of intraseasonal wind forcing in the simulations of both CR and CR_NoANIs_, we have performed two additional experiments (CR′ and ${{\rm{C}}{\rm{R}}^{\prime} }_{{\rm{NoANIs}}}$), by applying 150 day low-pass Butterworth filter (4’th order) on wind velocity. These ideal experiments are also used to understand the role of ocean internal instabilities in the formation of eddies in the BoB. They confirm that more than 96% of mesoscale eddies are having life cycles between ~28 days to ~150 days. We have also found significant reduction in number of eddies (~29%) in ${{\rm{C}}{\rm{R}}^{\prime} }_{{\rm{NoANIs}}}$ compared to CR′ in the WBoB (Fig. S6). This implies that ocean internal instabilities dominate in the eddy formation at the above intraseasonal time scale.

We have found similar reduction in the total number of eddies in the ${{\rm{C}}{\rm{R}}^{\prime} }_{{\rm{NoANIs}}}$ compared to CR′ only during winter season in the WBoB, but not during summer season (Fig. 2c–e). During winter season, total number of eddies decreases from 434 to 232 (~50%) in the absence of ANIs in our internal instability experiments. At all three locations in the WBoB, similar changes have been observed between instability experiments (CR′ − ${{\rm{C}}{\rm{R}}^{\prime} }_{{\rm{NoANIs}}}$) and the control simulations (CR − CR_NoANIs_). This implies that the decrease in ocean instabilities in the absence of ANIs plays a dominant role in the reduction of number of eddies in the WBoB during winter season. However, no significant change in the number of eddies has been observed during summer seasons associated with the instabilities produced by the presence of ANIs in the WBoB (Fig. 2 and Table 1). We found opposite characteristic in our instability experiments (CR′ − ${{\rm{C}}{\rm{R}}^{\prime} }_{{\rm{NoANIs}}}$) compared to control (CR − CR_NoANIs_) simulations at all the three locations of the WBoB during summer season. While SWBoB show a decrease in number of eddies from CR to CR_NoANIs_, an increase in number of eddies is observed from CR′ to ${{\rm{C}}{\rm{R}}^{\prime} }_{{\rm{NoANIs}}}$. A similar opposite response of the ANIs can be seen at CWBoB and NWBoB as well. This suggests that, during summer season, altered instability owing to the absence of ANIs do not contribute to the observed decrease in the number of eddies in the WBoB (484 to 289). On the other hand, it is likely to be associated with the oceanic response to the local intraseasonal winds and winds from the EIO. Due to the dominance of intraseasonal wind, we have observed an increase (512 to 661) in number of eddies in the ResBoB (Fig. 2b). This increase compensates the decrease in the number of eddies in the WBoB and hence almost no significant change is observed (996 to 960) in the number of eddies in the entire BoB during summer season in the absence of ANIs (Fig. 2a). However, during winter season due to dominance of ocean internal instability, a significant decrease (1091 to 659) is observed in the entire BoB in the absence of ANIs. This significant reduction in the total number of eddies are associated with cyclonic eddies in the WBoB compared to anticyclonic during winter seasons (Fig. 2a,c–e and Table 1). Based on our ocean internal instability experiments, we find that the cyclonic number of eddies decreases from 335 to 77 (decrease of ~77%); however, for anticyclonic eddies, a small increase (99 to 155) is observed during winter season in the WBoB (Table 1 and Fig. 2c–e).

Several previous studies have confirmed the role of barotropic and baroclinic instabilities on mesoscale eddy formation in the BoB^6,7,11,12^. We have used CR′ and ${{\rm{C}}{\rm{R}}^{\prime} }_{{\rm{NoANIs}}}$ for our eddy energy and instability analysis. We have estimated Eddy Kinetic Energy (EKE) and Eddy Potential Energy (EPE) for mean to eddy flow conversion in the BoB. EKE is defined as

1 $${\rm{EKE}}=\frac{1}{2}(u{^{\prime} }^{2}+v{^{\prime} }^{2});$$

and EPE is defined as

2 $${\rm{EPE}}=-\,\frac{g\rho ^{\prime} }{2\rho (\partial {\bar{\rho }}_{\theta }/\partial z)}.$$

${\rho }_{\theta }$ is the seasonal and horizontal mean potential density within the BoB; $\rho ^{\prime} =\rho -\bar{\rho }$, $u^{\prime} =u-\bar{u}$, and $v^{\prime} =v-\bar{v}$; $\bar{\rho }$ is the seasonal mean density and $\bar{u}$ and $\bar{v}$ are the zonal and meridional components, respectively of the seasonal mean current. As was done in Mukherjee *et al*.^11^, here also we use a cutoff of 151 days to define the seasonal mean.

Both EKE and EPE are generated in the ocean due to instability. Earlier studies have showed the evidences of both barotropic (*E*_BT_) and baroclinc (*E*_BC_) instabilities in the BoB. The estimation of *E*_BT_ and *E*_BC_ are based on^7,11,19,20^ and are given below

3 $${E}_{{\rm{BC}}}=-\,\frac{g}{\rho (\partial {\rho }_{\theta }/\partial z)}(u^{\prime} \rho ^{\prime} \frac{\partial \bar{\rho }}{\partial x}+v^{\prime} \rho ^{\prime} \frac{\partial \bar{\rho }}{\partial y}),$$

and

4 $${E}_{{\rm{BT}}}=-\,[u^{\prime} u^{\prime} \frac{\partial \bar{u}}{\partial x}+u^{\prime} v^{\prime} (\frac{\partial \bar{v}}{\partial x}+\frac{\partial \bar{u}}{\partial y})+v^{\prime} v^{\prime} \frac{\partial \bar{v}}{\partial y}]\mathrm{.}$$

Positive values of *E*_BC_ (*E*_BT_) indicate baroclinic (barotropic) conversion from mean to eddy flow.

**Figure 3:**
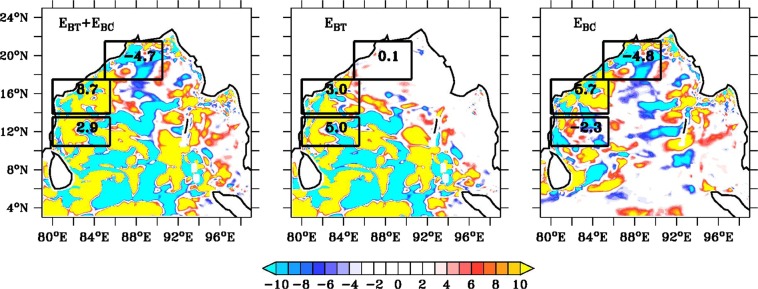
Winter (October–January) climatology of instabilities based on difference between CR′ and ${{\rm{C}}{\rm{R}}^{\prime} }_{{\rm{NoANIs}}}$. Left, middle and right panel shows combination of both barotropic and baroclinic ((E_*BT*_ + E_*BC*_, 10^−3^ m^2^ s^−3^), barotropic (E_*BT*_, 10^−3^ m^2^ s^−3^) and baroclinic (E_*BC*_, 10^−3^ m^2^ s^−3^) instability difference between above two models. Number in each square box denotes mean value of respective domain. Seasonal climatology of eddy energy due to instabilities of ANIs is shown in Fig. S7. Seasonal climatology of instability due to ANIs during spring and summer is shown in Fig. S8. Black contour represents land-sea masking based on Etopo20.

We have created a seasonal (spring, summer and winter) climatology of both the conversion terms for the January 2011–December 2015 to understand the role of instabilities in the BoB. We have found an increase (increase in total eddy energy, which is a combination of EKE and EPE, is shown in Fig. S7) in total instability (*E*_BT_ and *E*_BC_ combine) using differences of CR′ and ${{\rm{C}}{\rm{R}}^{\prime} }_{{\rm{NoANIs}}}$ simulations during winter in SWBoB (average value of +2.9 × 10^−3^ m^2^ s^−3^) and in CWBoB (average value of +8.7 × 10^−3^ m^2^ s^−3^) (Fig. 3). This implies the formation of mean to eddy flow conversion due to ANIs during winter season at CWBoB and SWBoB. This further suggests that the decrease in the total number of eddies in the CWBoB and SWBoB during winter due to the absence of ANIs are related to eddy formation via both *E*_BT_ and *E*_BC_ in the presence of ANIs. During winter at NWBoB, change of instability due to presence of ANIs is negative (mean value of −4.7 × 10^−3^ m^2^ s^−3^) and is responsible for increase in total number of eddies from 59 to 85. This above increase at NWBoB is associated with baroclinic conversion from eddy to mean flow (mean value −4.8 × 10^−3^ m^2^ s^−3^). However, increase at NWBoB is much weaker compared to decrease in number of eddies at CWBoB (237 to 117) and SWBoB (138 to 30) due to instability formation using ANIs during winter season. Due to dominance of positive instability in the WBoB, we have found significant reduction (434 to 232) in number of eddies during winter seasons in the absence of ANIs.

### Impact on coastal circulations

**Figure 4:**
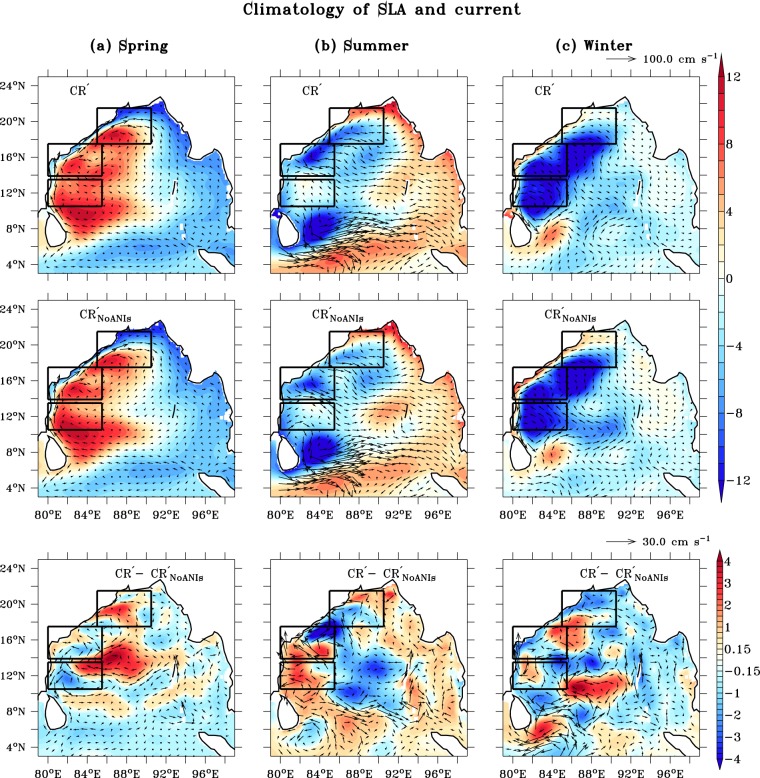
Seasonal climatology of SLA (cm) and current (cm s^−1^) using CR′ (top panel), ${{\rm{C}}{\rm{R}}^{\prime} }_{{\rm{NoANIs}}}$ (middle panel) and CR′ − ${{\rm{C}}{\rm{R}}^{\prime} }_{{\rm{NoANIs}}}$ (bottom panel) during spring, summer and winter seasons, respectively. Seasonal climatology of SLA and current using CR and CR_NoANIs_ is shown in Fig. S5.

It is fair to assume that the barotropic instability in the WBoB owing to the presence of ANIs should be associated with the changes in horizontal gradient of coastal current^20^. During spring season, positive SLA gradient (which leads to poleward EICC) is observed using both CR′ and ${{\rm{C}}{\rm{R}}^{\prime} }_{{\rm{NoANIs}}}$ (first and second column of Fig. 4a). However, very weak and negligible changes in both SLA and currents are observed during spring seasonin the ${{\rm{C}}{\rm{R}}^{\prime} }_{{\rm{NoANIs}}}$ simulation compared to CR′ (third column of Fig. 4a). This is related to weak instability formation in the presence of ANIs during spring season compared to summer and winter in the WBoB (Figs S8 and 3). Maximum change for SLA and currents in the absence of ANIs has been observed during both summer and winter (Fig. 4b,c).

During summer, ${{\rm{C}}{\rm{R}}^{\prime} }_{{\rm{NoANIs}}}$ increases (decreases) strength of the negative SLA (positive offshore SLA gradient) particularly in the CWBoB compared to CR′. Weakening of positive SLA gradient during summer season in the absence of ANI is responsible for increase EICC strength in the CWBoB using CR and also change of EICC direction from north-eastward using ${{\rm{C}}{\rm{R}}^{\prime} }_{{\rm{NoANIs}}}$ to northward using CR′ (Fig. 4b). During winter season at CWBoB, the case is opposite (Fig. 4). ${{\rm{C}}{\rm{R}}^{\prime} }_{{\rm{NoANIs}}}$ increases strength of the both positive SLA and negative offshore SLA gradient during winter season along western boundary of the BoB. Strengthening of positive SLA due to absence of ANI is responsible for weak EICC magnitude using CR′ in the south-west direction during winter seasons compared to ${{\rm{C}}{\rm{R}}^{\prime} }_{{\rm{NoANIs}}}$.

## Summary and Discussions

In this manuscript, we have discussed contributions of ANIs in mesoscale eddy (amplitude, radius and life cycle grater than 4 cm, 50 km and 28 days respectively) formation in the entire BoB between January 2011–December 2015. Our analysis shows significant reduction in total number of eddies in the WBoB in the absence of ANIs (~32%, ~20% and ~56% for NWBoB, CWBoB and SWBoB, respectively). Decrease in total number of eddies in the WBoB are associated with cyclonic eddies compared to anticyclonic.

During spring seasons, the performance of our CR is not good in simulation of altimeter observed number of eddies and produce much less eddy compared to summer and winter seasons. It is well known that circulation in the BoB is strongly modified by westerly wind bursts during November–December and April–May (Fig. S9), which are known to force strong eastward currents, also known as Wyrtki Jets^21,22^. These eastward currents then radiate downwelling equatorial Kelvin from the forcing region^22–26^. As this equatorial downwelling waves reaches the eastern boundary of the basin, reflect as a packet of coastally trapped waves and Rossby waves at the coast of Sumatra. These reflected waves then propagate along the eastern boundary, and across the interior, of the Andaman Sea, eventually radiate out as downwelling Rossby waves and impacting circulations around the ANIs and in the interior of the BoB^10,27–30^. The theoretical speed of Rossby wave can be estimated using $\beta {c}_{n}^{2}/{f}^{2}$ (McCreary *et al*.^31^), where, $\beta =\partial f/\partial y$ (equatorial beta plan approximation), *f* is the Coriolis parameter and *c*_*n*_ is the speed of n’th order baroclinic mode Kelvin wave. For first mode, typical value of *c*_*n*_ is ~264 cm s^−1^ (Mukherjee *et al*.^11^). The value of *β* is 1.97 × 10^−13^ cm^−1^ s^−1^ at EIO. *f* can be estimated using $2\omega$ sin *y*, where *y* is the latitude angle and $\omega$ is the angel of earth rotation (7.3 × 10^−5^ s^−1^). According to above formulation, the speed of Rossby wave will be ~6.2 cm s^−1^ at 15°N. So, reflected Rossby wave will take ~2–3 months from eastern boundary of the BoB to the WBoB (~90°E–85°E) and influence the circulation there. Hence, inability of the model to simulate accurate phase/speed of the remotely forced Rossby waves is the likely cause for the weaker eddy activity during spring season in the model.

Recent studies show that there is year-to-year variability in the strength of Wyrtki Jets during both above months and this interannual variability is found to be associated with El Niño- Southern Oscillation (ENSO^32^), Indian Ocean Dipole (IOD^33^) and Madden Julian Oscillation (MJO^34^). It is known based on previous research of Duan *et al*.^35^ that anomalous behavior of significantly stronger eastward Wyrtki Jet during December 2013 was associated with strong intraseasonal variability, known as MJO in the EIO. Previous researchers also showed weakening of westerly wind bursts during both November - December and April - May between 2006–2008 and evidences of weaker easterly wind burst during above months on some occasions^36^. Westward propagation of wind (easterly wind) along the EIO is responsible for the formation of upwelling Kelvin waves and associated upwelling Rossby waves in the eastern EIO^36^. In a recent study, Chen *et al*.^9^ showed the dominance of remote equatorial response on EKE along WBoB during spring season compared to summer and winter seasons. However, their study was limited in terms of climatology. A detailed study is required to understand the impact of year-to-year equatorial Wyrtki Jets variability in the formation of eddies in the BoB and associated interannual variability.

Maximum impact of ANIs in the WBoB due to ocean internal instability are observed during winter season compared to summer ans spring seasons. During summer seasons, intraseasonal wind (both from local BoB and remote EIO) play an important role in mesoscale eddy formation in the WBoB compared to instability produced by ANIs. However, during winter seasons, strong barotropic and baroclinic instability are formed in the both CWBoB and SWBoB due to presence of ANIs and increase eddy activity. Our study confirmed that, the significant decrease of the number of eddies during winter seasons at SWBoB (~50%) and CWBoB (~91%) in the absence of ANIs are associated with the formation of both above two types of instabilities driven by ANIs. Instability produced by ANIs in our model also shows significant impact in sea level and current variability along WBoB during summer and winter seasons compared to spring by changing mean to eddy flow conversion and vice-versa.

Change in the number of anticyclonic eddies in the absence of ANIs is not homogeneous along WBoB due to significant increase (decrease) at CWBoB (SWBoB). One possible reason of this heterogeneity may be associated with dominant change of anticyclonic eddies in the WBoB in the absence of ANIs during spring season at SWBoB and summer seasons at CWBoB compared to winter seasons (Fig. 2c,d), when the role of ocean internal instability driven by ANIs is maximum. As SWBoB is located in the southern part of the WBoB, impact of downwelling Rossby wave from easternRossby wave associated with strong eastward wind forced Wyrtki jet in the EIO is downwelling favorable and responsible for high SLA and deepening the thermocline in the BoB. boundary of the BoB during spring season will be significant. Due to this, we have observed significant decrease in anticyclonic number of eddies in the SWBoB during spring season in the absence of ANIs (Fig. 2c). However, at CWBoB, maximum change in anticyclonic number of eddies in the absence of ANIs is observed during summer season compared to spring and winter (Fig. 2d). During summer season, the internal instability analysis in the CWBoB (Fig. S8) shows negative mean value of instabilities (−14.3 × 10^−3^ m^2^ s^−3^) driven by ANIs, which further imply more mean to eddy (eddy to mean) flow conversion in the absence (presence) of ANIs. However, due to dominance of strong mean current at CWBoB compared to SWBoB and NWBoB (Fig. S5), impact of instability driven by ANIs during summer season is less on anticyclonic eddies and likely be responsible for the dominance of decrease in number of cyclonic eddies in the CWBoB compared to increase in number of anticyclonic eddies.

Our study suggests that ANIs need to be accurately represented in an ocean or climate model due to its role in mesoscale eddy formation in the BoB. Previous studies confirmed the role of cyclonic eddy using upwelling in enhancing the biological productivity^3,4,37,38^ and marine fisheries^39^ along WBoB. Using our study, we have found a quantitative link between ANIs and ocean productivity along east coast of India. Our study also highlights thatANIs will play a critical role in regional climate of the North Indian Ocean (NIO) by changing eddy driven transport of heat and salt in the NIO, which need to be studied in future.

## Supplementary information

Supplementary pdf
