# Supplementary material for: Role of Andaman and Nicobar Islands in eddy formation along western boundary of the Bay of Bengal
Source: Sci Rep. 2019 Jul 12;9:10152. doi: 10.1038/s41598-019-46542-9 (PMC6625997; doi:10.1038/s41598-019-46542-9)
Supplement: Supplementary file 1 — Supplementary pdf [file 41598_2019_46542_MOESM1_ESM.pdf]

Supplement to: Role of Andaman and Nicobar Islands in eddy formation along western boundary of the Bay of Bengal

A. Mukherjee[1,*], Abhisek Chatterjee[1], and P. A. Francis[1]

[1]Indian National Centre for Ocean Information Services (INCOIS), Hyderabad, India.
[*]amukherjee.physics@gmail.com

S1 Eddy detection algorithm

We have used an eddy detection algorithm on gridded sea level anomaly (SLA) based on Mason et al[1].

Eddy identification

We first removed high frequency noises from SLA using Gaussian filter, which is applied spatially with a zonal (meridional) major (minor) radius of 10° (5°). In our domain of interest (3°–23°N and 78°–98°E), SLA contours are computed between -100 to 100 cm levels with 1 cm intervals. To identify cyclonic (anticyclonic) eddies, the contours are searched from 100 (-100) cm downward (upward). At each SLA interval, closed contours (CC) are sequentially identified and analysed. For selection of a potential eddy, CC will meet five criteria.

First criteria is to pass a shape test with error $\leqslant 55\%$, which is defined as the ratio between the areal sum of CC deviations from its fitted circle and the area of that circle. Second criteria using CC is related with satisfying minimum (8) and maximum (100) pixel count. Third criteria is that CC should contain only pixels with SLA values above (below) the present SLA interval value for anticyclonic (cyclonic) eddies. Fourth criteria is that CC will never contain more than one local maximum (minimum) for an anticyclonic (cyclonic) eddy. Fifth criteria is that amplitude of CC (A) will be limited between 4 and 150 cm.

If CC satisfy all above five criteria, then it is identified as an eddy with effective perimeter (Peff). Similarly, an effective eddy radius (Reff) is defined based on Peff. Next a speed based eddy radius (Rspeed) is found using the radius of the circle with same area as the region within the CC of SLA with maximum average geostrophic speed. Eddy radius (Reddy) is estimated by iterating from Reff inward over all CC, whose pixel count are between 8 and 100. At the end, SLA pixels corresponding to the eddy marked and make unavailable for further eddy identification.

Eddy tracking

Eddy tracking (Etrack) is performed using centroid corresponds to the contour of last iteration using CC. Tracking of cyclonic and anticyclonic eddies are performed separately. Difference in distances between all identified Etrack at time steps k and k+1 are computed. Connection of k+1 eddies to k is decided using ellipse method discussed in Mason et al[1]. The coordinates of Etrack and parameters Reff, A, Rspeed etc. are stored for both cyclonic and anticyclonic eddies with lifetime grater than 28 days.

S2 Ocean model

The topography of our ROMS model (referred as CR in the manuscript) is based on 2-minute resolution bathymetry using Sindhu et al[2]. The narrow channel between India and Sri Lanka remains closed in the model and isolated land points are removed. Moreover, we closed the shallow Mallacca strait and thus, the part of the South China Sea is also removed from model grid. No slip and no normal flow boundary conditions are applied on the northern and western continental land boundaries. However, for the southern and eastern open boundaries, the momentum and tracer fields are nuddged with the global solutions from INCOIS-GODAS[3] with a time scale of 5 days. INCOIS-GODAS is a global ocean model based on Modular Ocean Model version 4.0 (MOM4p0) and three dimensional variational (3D-VAR) data assimilation scheme. Detailed of open boundary conditions are provided in Table 1.

We have used second order, centered, finite difference scheme for advection of momentum and tracer. The sub-grid scale for dissipation of momentum are based on Wajsowicz (1993)[4]. The shortwave penetration scheme of Paulson and Simpson

Lateral boundary condition	East	West	North	South	References
Free surface	Chapman	closed	closed	Chapman	[9]
2D U-momentum	Flather	closed	closed	Flather	[10]
2D V-momentum	Flather	closed	closed	Flather	[10]
3D U-momentum	Radiation	closed	closed	Radiation	[11]
3D V-momentum	Radiation	closed	closed	Radiation	[11]
Turbulent kinetic energy	Reduced physics	closed	closed	Reduced physics	[10]
Temperature	Radiation	closed	closed	Radiation	[11]
Salinity	Radiation	closed	closed	Radiation	[11]

TableS 1. Lateral boundary conditions used in our ROMS model are listed with references.

.

(1997)[5] has been used for chlorophyll concentration in the upper ocean which includes double exponential profile for shortwave radiation absorption. We have used K-profile parametrization scheme (KPP) for vertical mixing[6]. Vertical mixing background coefficient for momentum and tracer are set to 1 x 10^{-6} and 1 x 10^{-5} m^2 s^{-1}, respectively. Laplacian horizontal viscosity coefficient for momentum is set to 500 m^2 s^{-1}. To include the effect of the river runoff implicitly in the model, the sea surface salinity is restored to the monthly climatology values from North Indian Ocean Atlas[7].

ROMS is initialized using the solution (horizontal currents, temperature, salinity and SLA) for January 1, 2010 from INCOIS-GODAS and then forced by the 6 hourly atmospheric fields (air pressure at 2m, air temperature at 2m, net shortwave radiation at 2m, net longwave radiation at 2m, relative humidity at 2m, precipitation at ocean surface, zonal wind speed at 10m, meridional wind speed at 10m) from Global Forecast System (GFS) at a horizontal resolution of 0.25°, obtained from National Centre for Medium Range Weather Forecasting (NCMRWF) (`http://www.ncmrwf.gov.in/gfs_report_final.pdf`;[8]).

S*References

1. Mason, E., Pascual, A. & McWilliams, J. C. A New Sea Surface Height–Based Code for Oceanic Mesoscale Eddy Tracking. *J. Atmos. Ocean. Technol.* **31**, 1181–1188 (2014).

2. Sindhu, B. *et al.* Improved bathymetric datasets for the shallow water regions in the Indian Ocean. *J. Earth syst. Sci.* **116 (3)**, 261–274 (2007).

3. Ravichandran, M. *et al.* Evaluation of the Global Ocean Data Assimilation System at INCOIS: The Tropical Indian Ocean. *Ocean. Model.* **69**, 123–135, DOI: http://dx.doi.org/10.1016/j.ocemod.2013.05.003 (2013).

4. Wajsowicz, R. C. A consistent formulation of the anisotropic stress tensor for use in models of the large scale ocean circulation. *J. Comput. Phys.* **333-338** (1993).

5. Paulson, C. A. & Simpson, J. J. Irradiance measurements in the upper ocean. *J. Phys. Oceanogr.* **952-956** (1977).

6. Large, W. G., McWilliams, J. C. & Doney, S. C. Oceanic vertical mixing: A review and a model with a nonlocal boundary layer parameterization. *Rev. Geophys.* **32(4)**, 363–403 (1994).

7. Chatterjee, A. *et al.* A new atlas for temperature and salinity for north Indian Ocean. *J. Earth Syst. Sci.* **121 (3)**, 559–593 (2012).

8. Prasad, V. S., Mohandas, S., Gupta, M. D., Rajagopal, E. N. & Dutta, S. K. Implementation of upgraded global forcasting systems (T382L64 and T574L64) at NCMRWF. *Tech. Report.* **NCMR/TR/5/2011**, National Centre for Medium Range Weather Forcast, New Delhi (2011).

9. Chapman, C. D. Numerical treatment of cross-shelf open boundaries in a barotropic coastal ocean model. *J. Phys. Ocean.* **15**, 1060–1075 (1985).

10. Flather, R. A. A tidal model of the northwest European continental shelf. *Memoires de la Soc. Royale de Sci. de Liege* **6**, 141–164 (1976).

11. Orlanski, I. A simple boundary condition for unbounded hyperbolic flows. *J. Comp. Sci.* **21(3)**, 251–269 (1976).

12. Han, W. & Webster, P. Forcing mechanisms of sea level inter-annual variability in the Bay of Bengal. *J. Phys. Ocean.* **32**, 216–239, DOI: 10.1175/1520-0485(2002)032<0216. (2002).

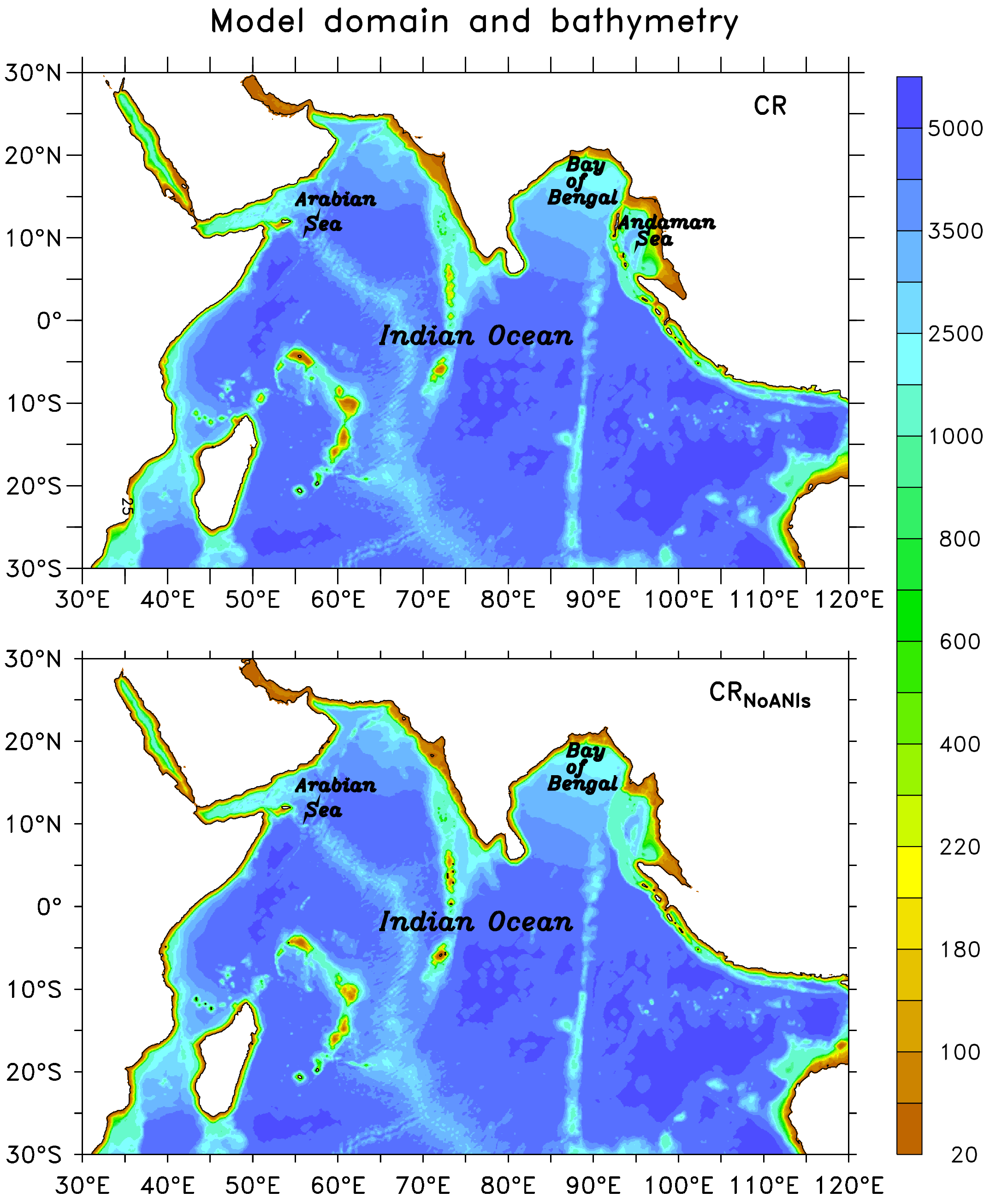

FigureS 1. Model domain with bathymetry for CR (top panel) and CR$_{NoANIs}$ (bottom panel).

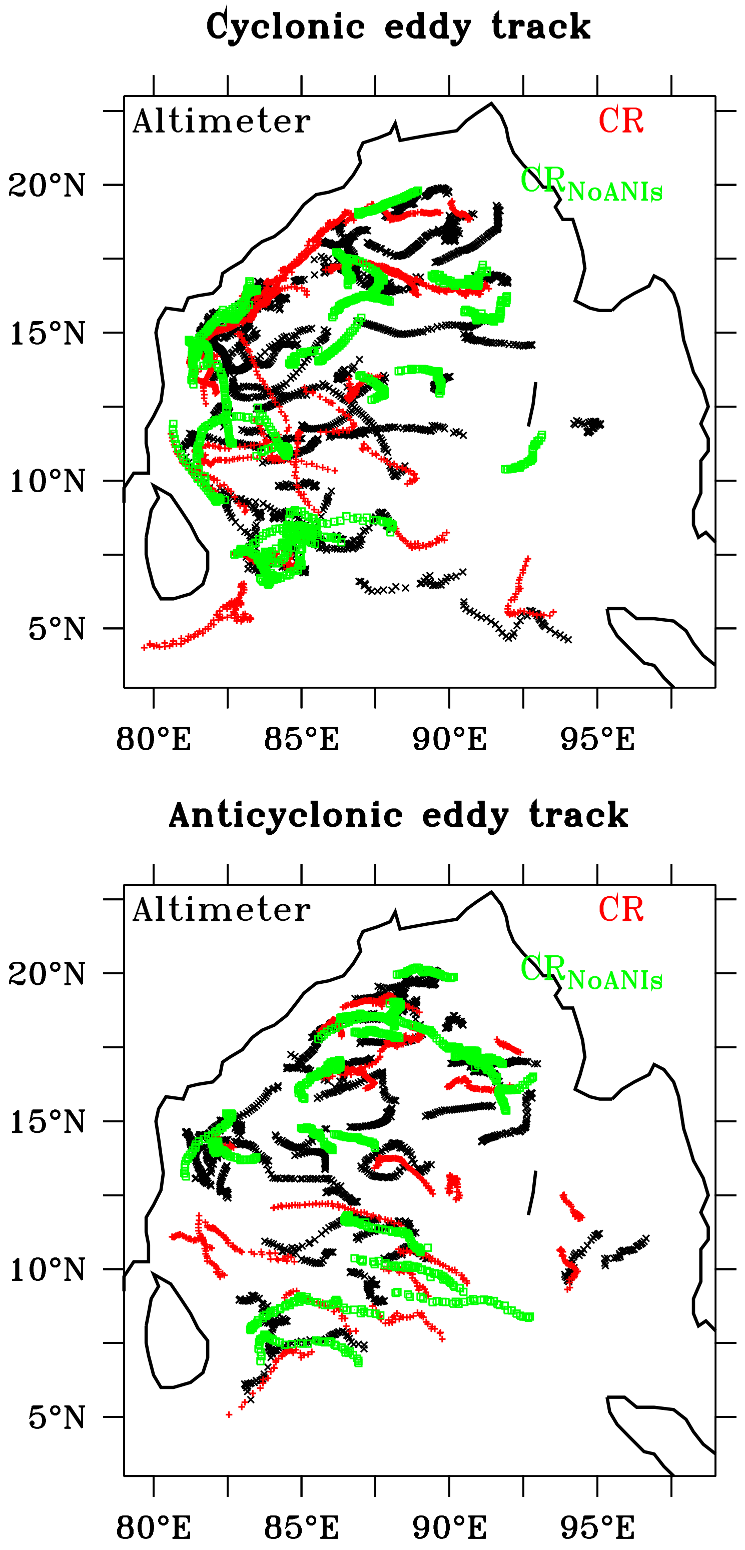

FigureS 2. Comparison of eddy track for cyclonic (top panel) and anticyclonic eddies (bottom panel) using altimeter (black colour), CR (red colour) and CR$_{NoANIs}$ (green colour).

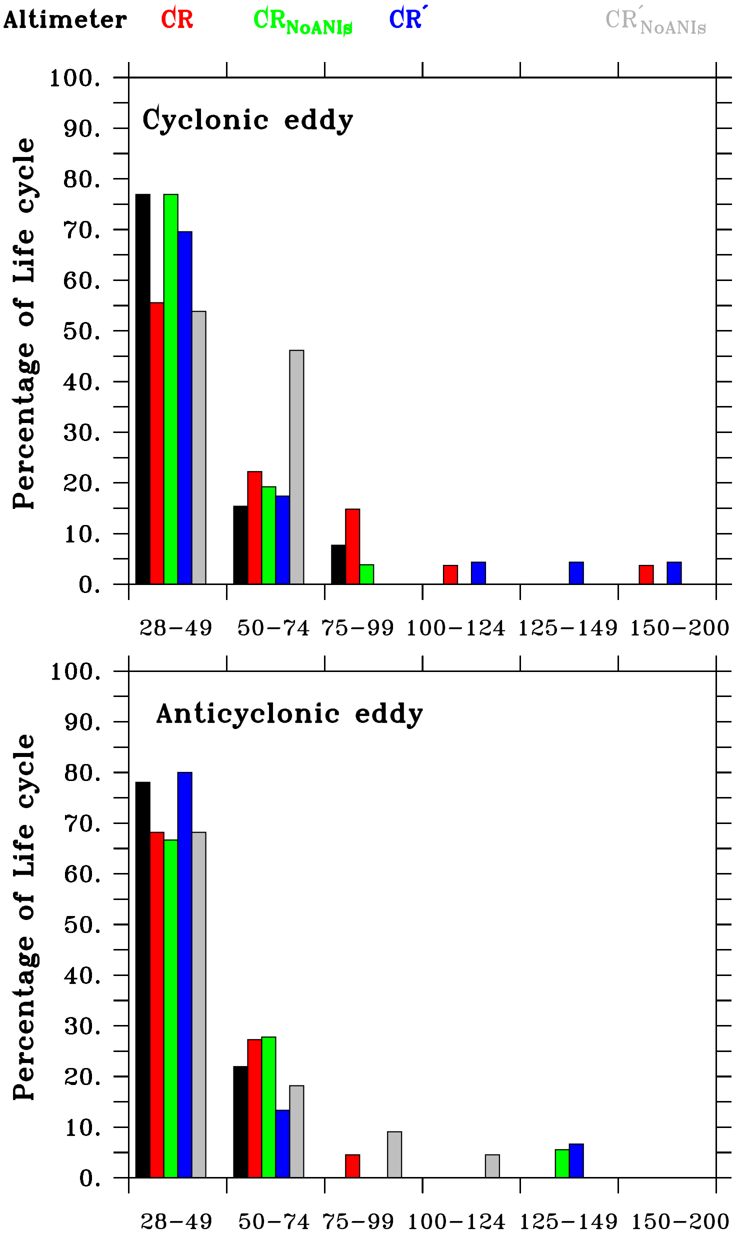

FigureS 3. Histogram of cyclonic (top panel) and anticyclonic eddies (bottom panel) related to percentage contribution of life cycle of eddies in days. Vertical (horizontal) axis shows percentage value (life cycle of eddies). Detailed of model simulations are discussed in the main manuscript.

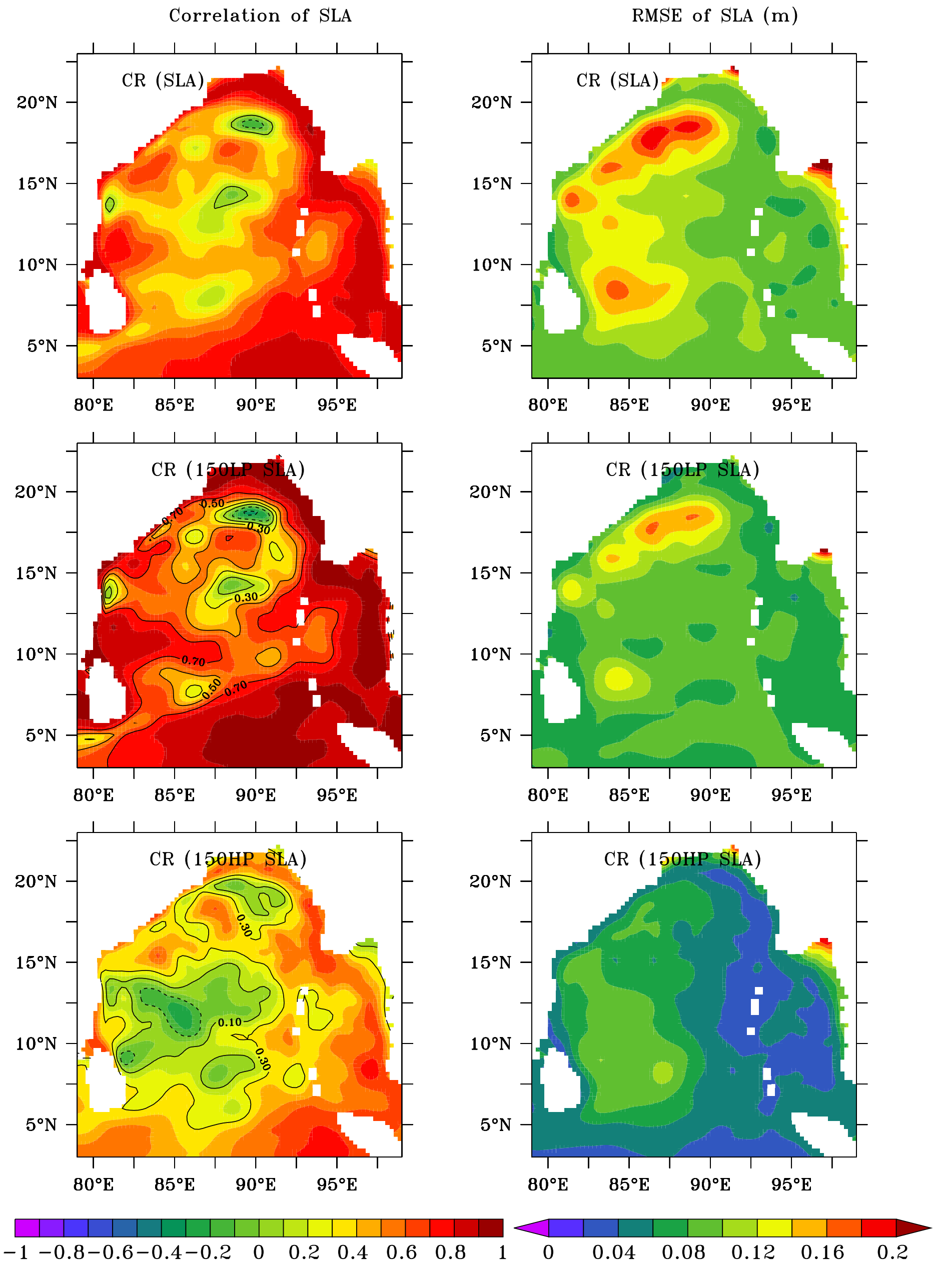

FigureS 4. Left (right) panel of the figure shows correlation (RMSE; root mean square error) of sea level anomaly (SLA) between altimeter and CR during 01-January-2011 to 31-December-2015. Top, middle and bottom panel of the figure represents SLA with no-filtered, 150-day low-pass time series and 150-day high-pass time series filtered SLA. Black contour line in left panel shows correlation values with less than 95% significant.

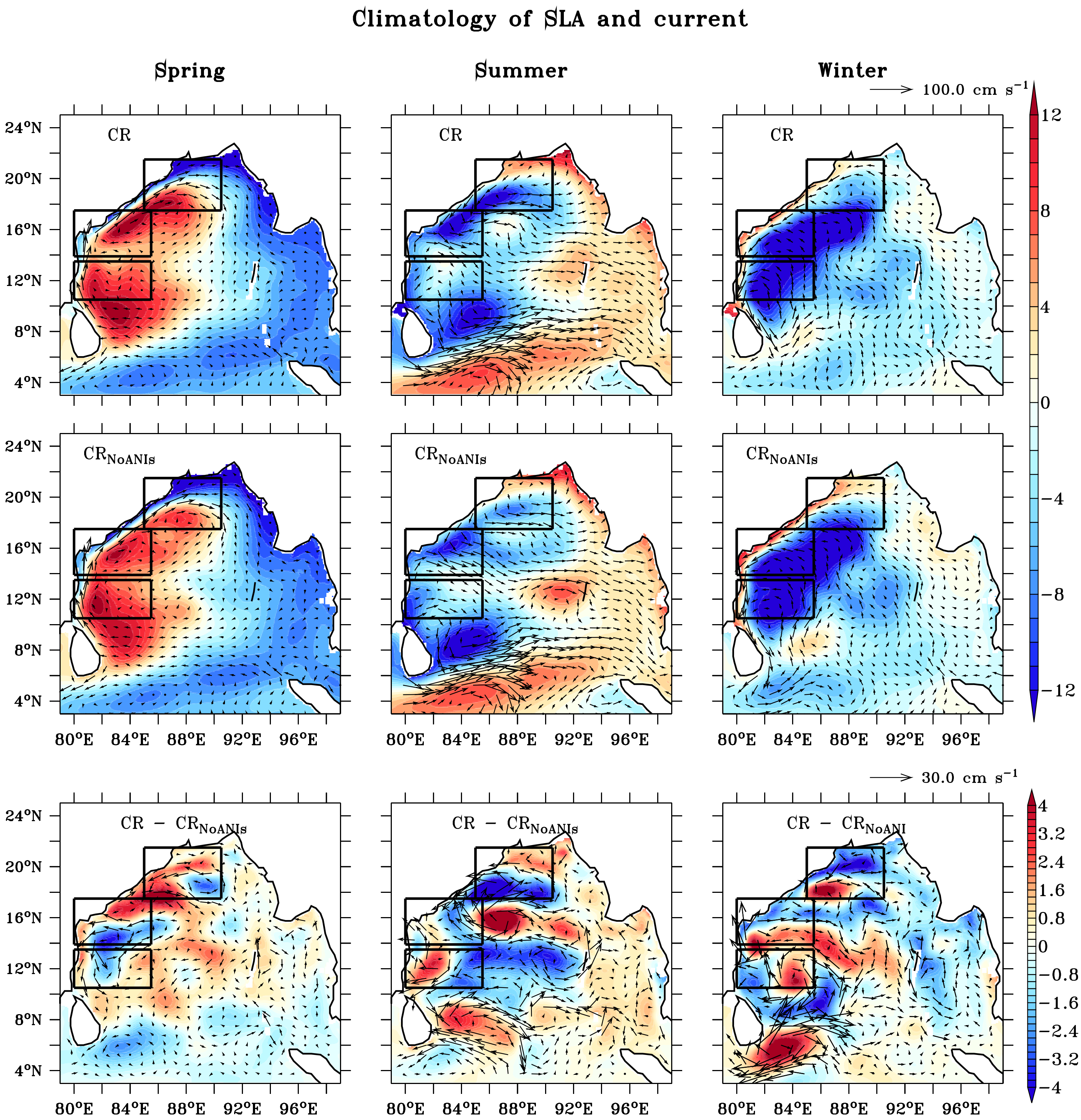

FigureS 5. Seasonal climatology of SLA (cm) and current (cm s^{-1} using CR (top panel), CR_{NoANIs} (middle panel) and CR - CR_{NoANIs} (bottom panel) during spring, summer and winter seasons. Detailed of model simulations are discussed in the main manuscript.

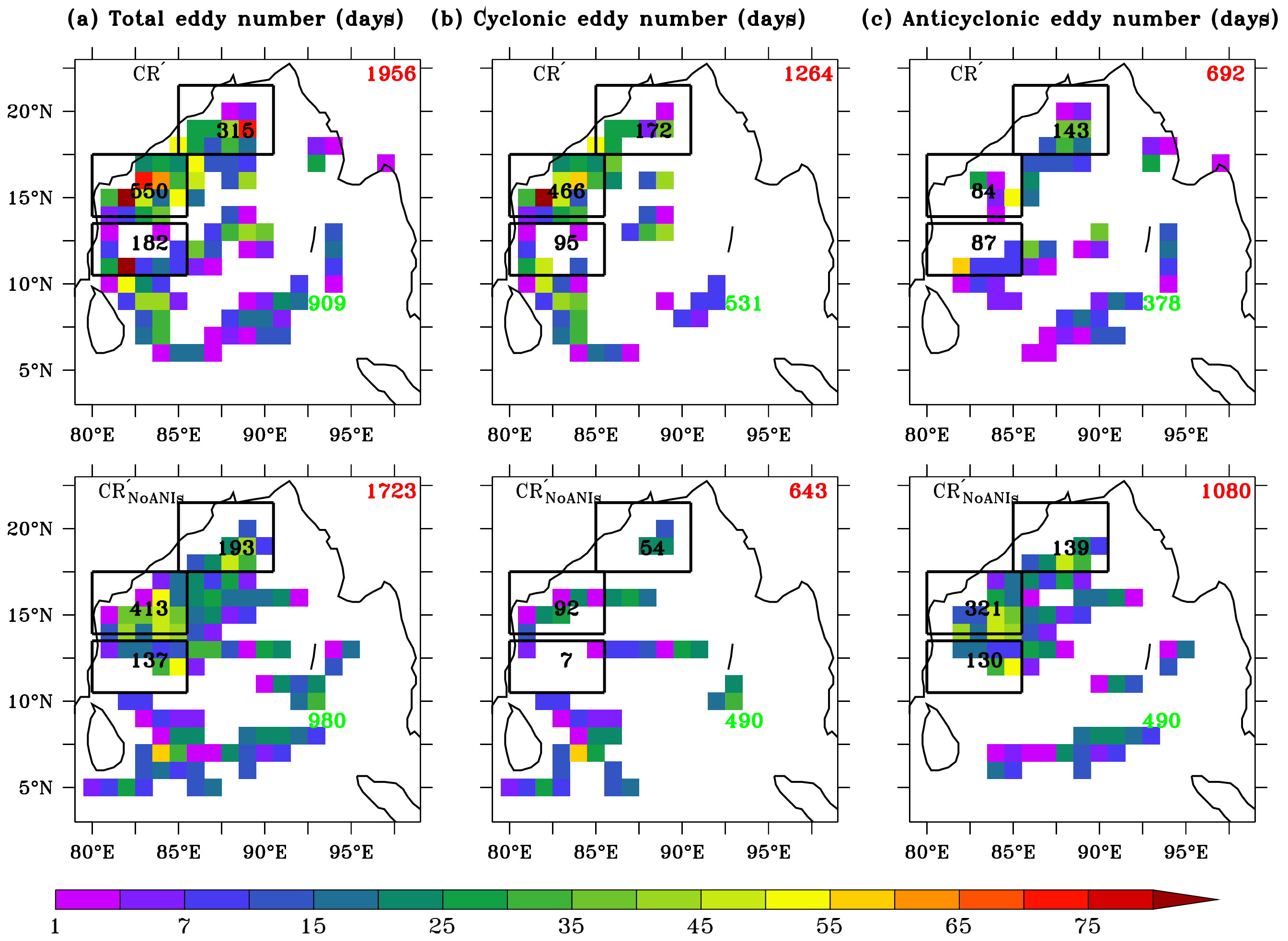

FigureS 6. Comparison of number of eddies (January 2011– December 2015) between two 150-day low-pass intraseasonal wind forcing experiments (CR′ and CR'_{NoANIs}) for total (combination of cyclonic and anticyclonic) (left panel), cyclonic (middle panel) and anticyclonic (right panel) number of eddies. The rectangular box between 80°–85°E and 10°–13°N, 80°–85°E and 14°–16°N and 85°–90°E and 17°–21°N denotes SWBoB, CWBoB and NWBoB respectively. Number denotes in three rectangular boxes represent sum of number of eddies in the respective region during above mentioned time period. The number in red (green) denotes sum of number of eddies in the entire BoB (ResBoB).

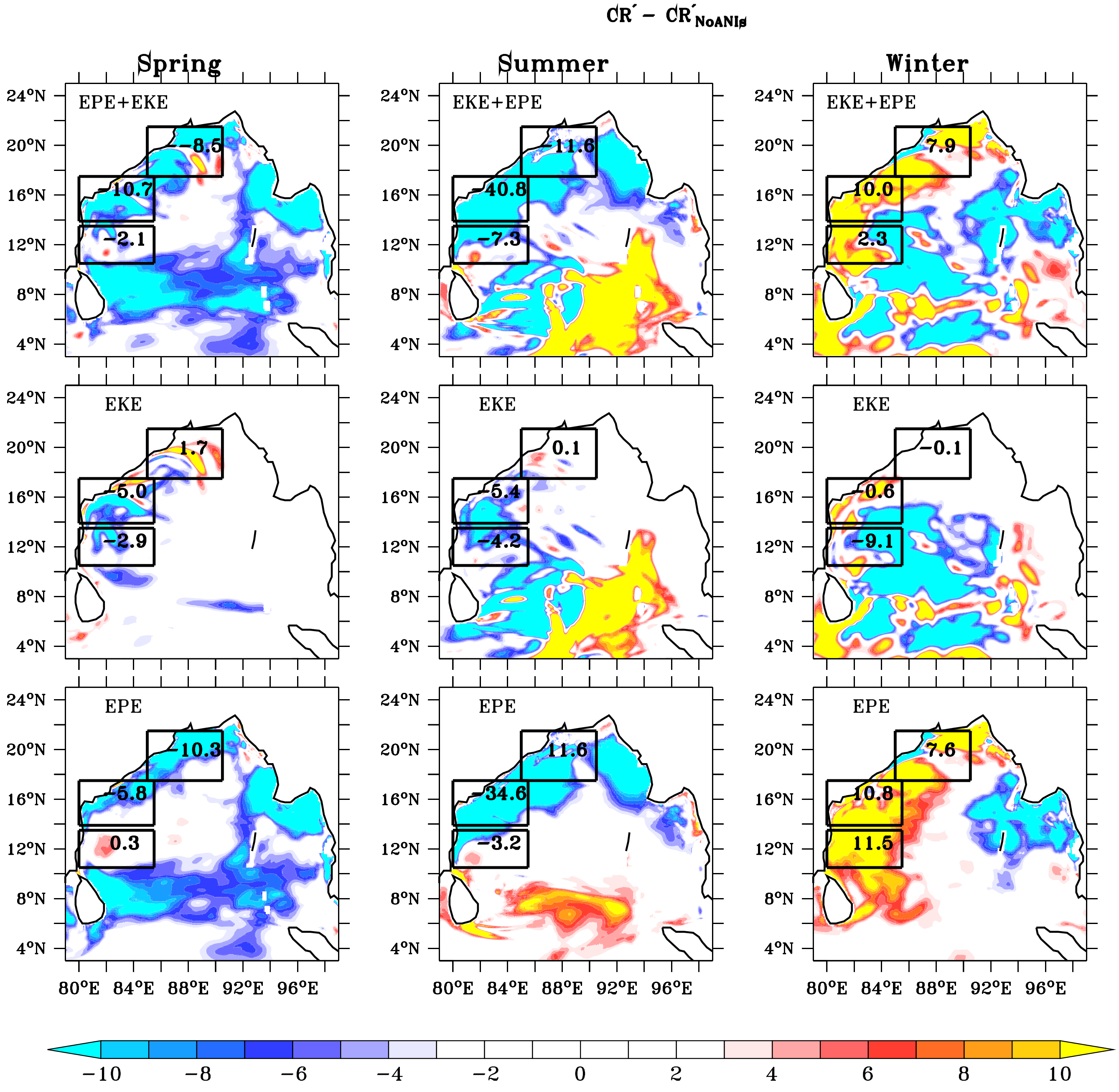

FigureS 7. Seasonal climatology of eddy energy based on difference between CR′ and CR'_{NoANI}. Top, middle and bottom panel shows combination of both eddy kinetic and potential ((EKE + EPE, 10^{-3} m^2 s^{-2}), eddy kinetic (EKE, 10^{-3} m^2 s^{-2}) and eddy potential, 10^{-3} m^2 s^{-2}) energy difference between above two models. Number in each square box denotes mean value of the respective domain.

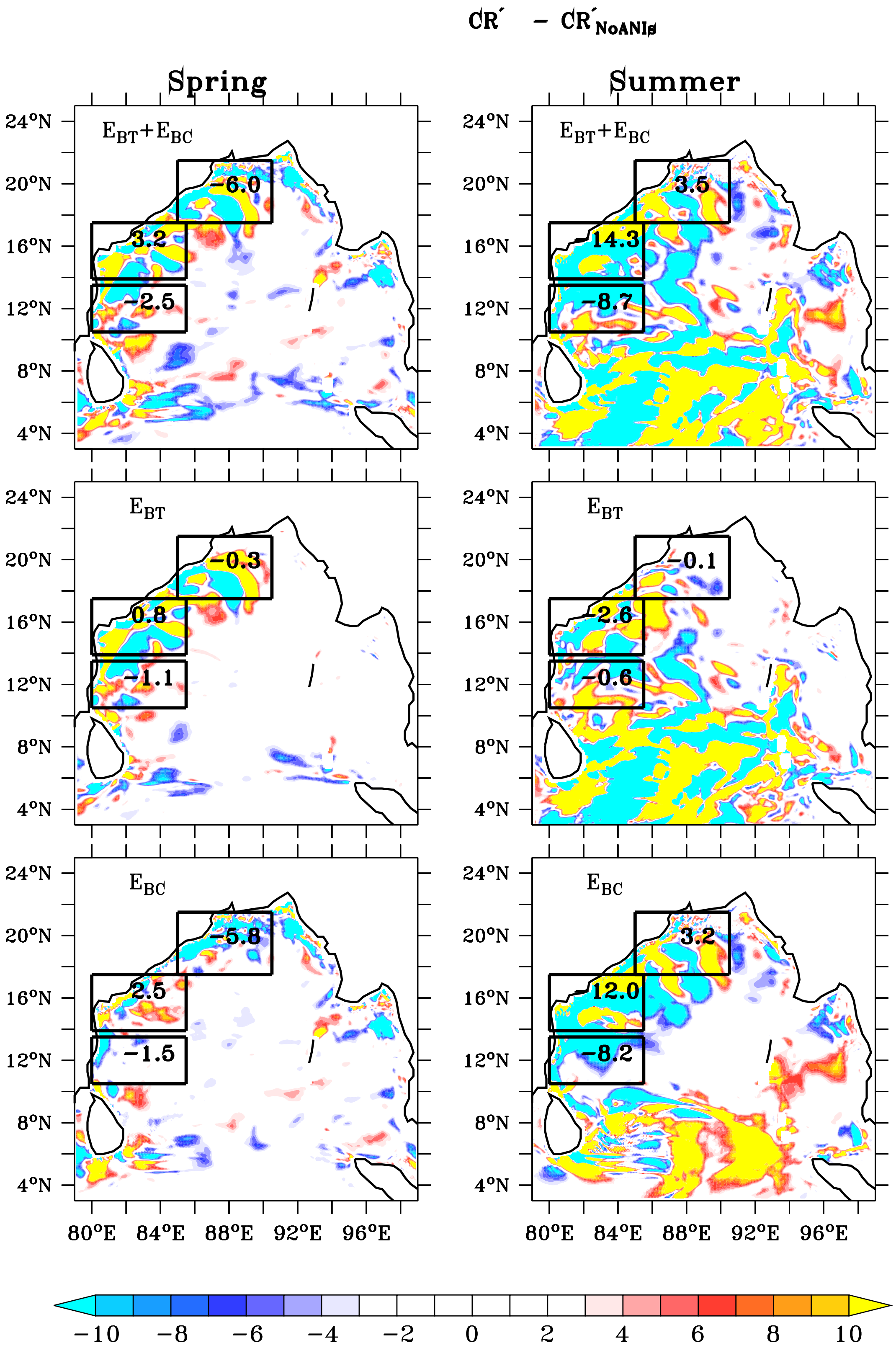

FigureS 8. Spring (February– May, left panel) and Summer (June–September, right panel) climatology of instabilities based on difference between CR′ and CR'_{NoANIs}. Top, middle and bottom panel shows combination of both barotropic and baroclinic ((E_{BT} + E_{BC}, 10^{-3} m^2 s^{-3}), barotropic (E_{BT} , 10^{-3} m^2 s^{-3}) and baroclinic (E_{BC}, 10^{-3} m^2 s^{-3}) instability difference between above two models. Number in each square box denotes mean value of respective domain. Seasonal climatology of eddy energy due to instabilities of ANIs are shown in Figure S7. Seasonal climatology of instability in the presence of ANIs during winter seasons is shown in Figure 3 of the main manuscript. Black contour represents land-sea masking based on Etopo20.

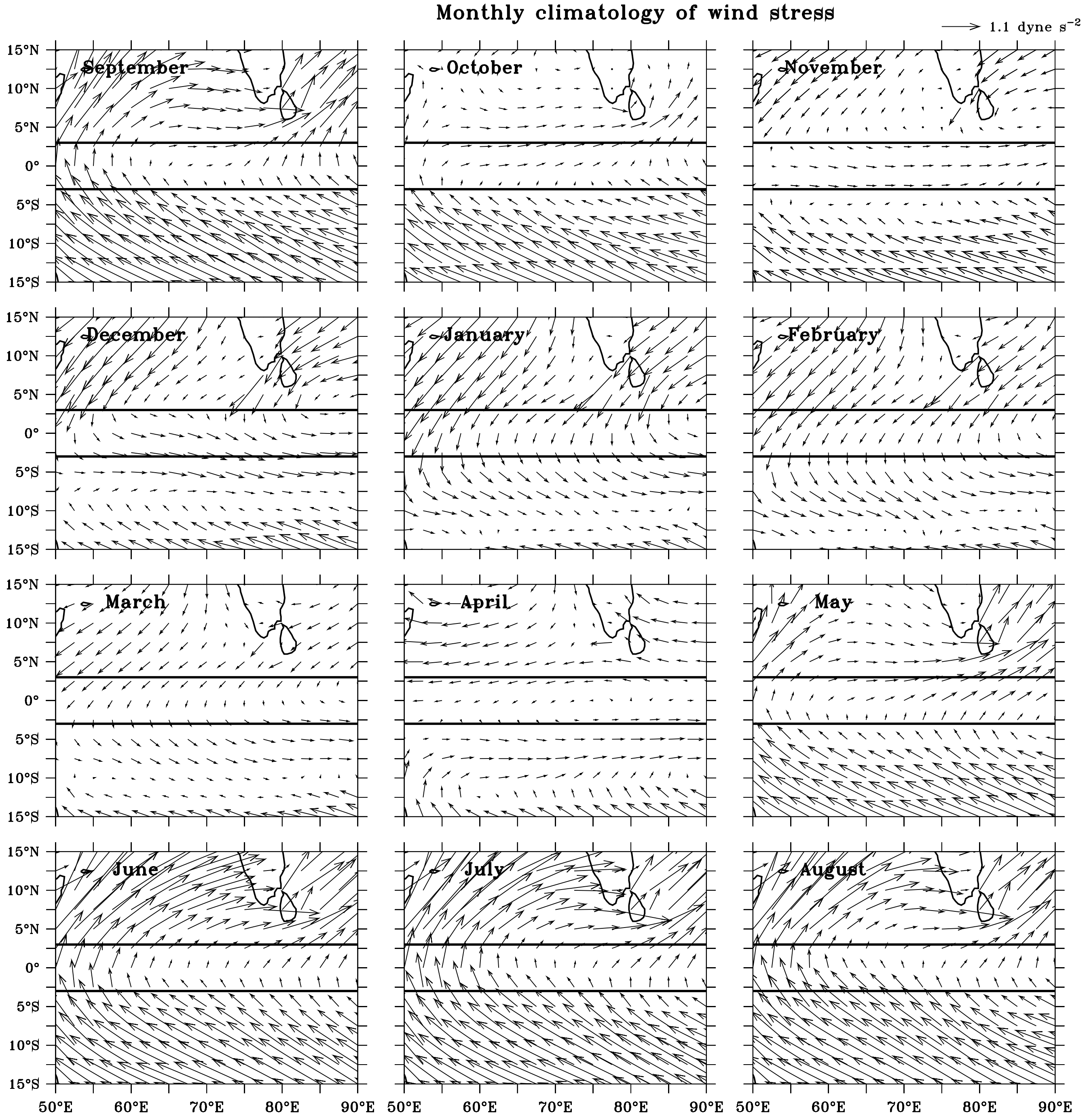

FigureS 9. Monthly climatology of NCMRWF wind stress (January 2010–December 2015) between 50°E–90°E/ 15°S–15°N. The estimation of wind stress from wind velocity are based on bulk formula described in Han and Webster[12].
